# Atypical Femoral Fractures: Implications for the Advanced Practitioner in Oncology

**Published:** 2017-05-01

**Authors:** Kathy Sharp

**Affiliations:** Wellmont Cancer Institute, Bristol, Virginia

## Abstract

Bone-health issues may arise for oncology patients as a side effect of their treatments. One of these may be the development of an atypical femoral fracture. Advanced practitioners should be aware of the risk factors for atypical femoral fractures, and be able to promptly recognize signs, provide patient education, and manage them competently.

Advanced practitioners in oncology (APs) frequently encounter bone-health issues in oncology. A significant number of oncology patients are at risk for accelerated bone loss due to treatment sequelae. Many of those patients may also have preexisting osteoporosis. Reports of atypical femoral fractures (AFFs) have created uncertainty about the duration of bisphosphonate or receptor activator of nuclear factor kappa-B ligand (RANKL) inhibitor therapy for bone health in both patients with cancer and osteoporosis, despite the fact that they are uncommon consequences of treatment for osteoporosis, osteopenia, and bony metastasis. The AP should be aware of the risk factors for AFF, be able to provide patient education, promptly recognize signs of an AFF, and manage it competently (Miller, 2010).

## HISTORY AND DEFINITION

Initially, AFFs were thought to be related to bone-turnover suppression and were likened to stress fractures. In researching the history of AFF, however, it was found that this type of fracture was recognized prior to the introduction of bisphosphonates.

There are many mechanisms behind AFF, and they are well documented in the biomechanics literature. A review in the Journal of Biomechanics stated that the mechanisms that lead to AFF have not been definitively identified, so a causal relationship between bisphosphonates and AFF has yet to be established (Geissler, Bajal, & Fritton, 2015; Velasco, Kim, Bleakney, & Jamal, 2014). Other researchers reported that patients with AFF had a longer duration of bisphosphonate use, as well as a higher body mass index and a higher total hip bone mineral density (Gedmintas, Solomon, & Kim, 2013). A 2013 meta-analysis of 11 studies in the Journal of Bone and Mineral Research concluded that bisphosphonate users had an increased risk of subtrochanteric, femoral shaft, and AFFs (Gedmintas et al., 2013).

A small study of 25 patients who had taken bisphosphonates for a mean duration of 9.84 years suggested suboptimal vitamin D levels may be a risk factor for development of AFF in addition to prolonged bisphosphonate use. AFFs are heralded by prodromal thigh pain in about 75% of cases (Markman et al., 2013; Mulcahy, 2014).

**Figure 1:**
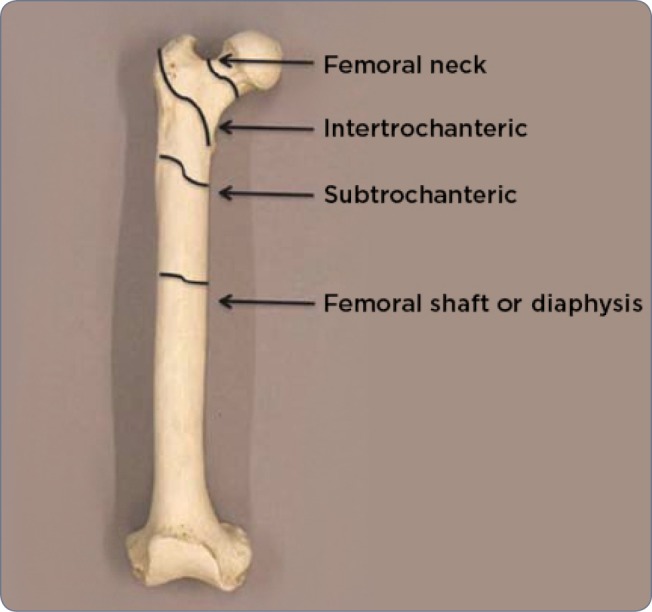
Locations of common hip and femur fractures. Image adapted from Shane et al. (2014).

The term "atypical femur fracture" was first described in 1978 and reported in publication in 2005. To settle ongoing confusion about what constituted an AFF, the American Society for Bone and Mineral Research Task Force published a position paper in 2010 to clarify what type of fractures were included, and this report was updated in 2014. Prior to that time, available research did not differentiate between subtrochanteric and femoral shaft fractures (Figure 1; Girgis & Seibel, 2011; Toro et al., 2016; Shane et al., 2014).

To be defined as an AFF, a fracture must meet the major criteria listed in Table 1. However, as for the minor features listed in Table 1, their presence/absence is not required for a fracture to be defined as an AFF.

**Table 1:**
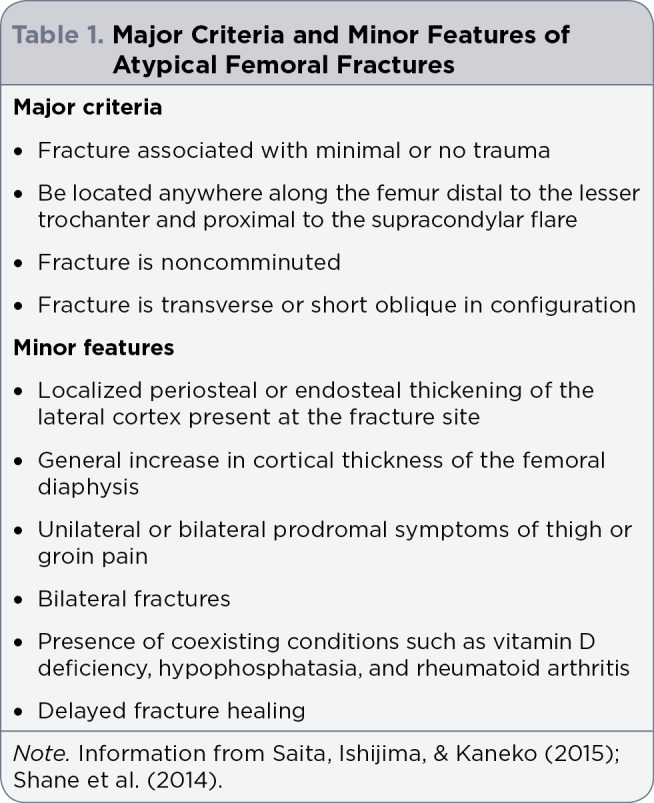
Major Criteria and Minor Features of Atypical Femoral Fractures

## INCIDENCE

Although there have been reports of AFF for more than 10 years, it is still not well understood, and the reports of its incidence vary greatly. The majority of accounts of AFF in the cancer population have been primarily in postmenopausal women treated for a prolonged period with a bisphosphonate. There are conflicting reports of AFF occurrence in patients taking denosumab (Xgeva).

The denosumab FREEDOM trial reported no incidence of AFF in the first 3 years of therapy. According to the American Society for Bone and Mineral Research (Shane et al., 2014), the relative risk of AFF is high, but the absolute risk is low, ranging from 3.2 to 50 cases per 100,000 person-years. By 2010, the US Federal Drug Administration (FDA) believed there was sufficient evidence to link the use of bisphosphonates and AFF, and announced a required labeling change for all bisphosphonates used to treat osteoporosis. Medications used to treat Paget’s disease and cancer-related hypercalcemia (i.e., zoledronic acid, etidronate, and tiludronate) were not included in this labeling change (Shane et al., 2014).

## WHO IS AT RISK?

The National Comprehensive Cancer Network (NCCN) summarized the risk factors for AFF in a 2014 report (Mulcahy, 2014), and additional risk factors have been identified by Toro et al. (2016), based on review of 137 articles (Table 2).

**Table 2:**
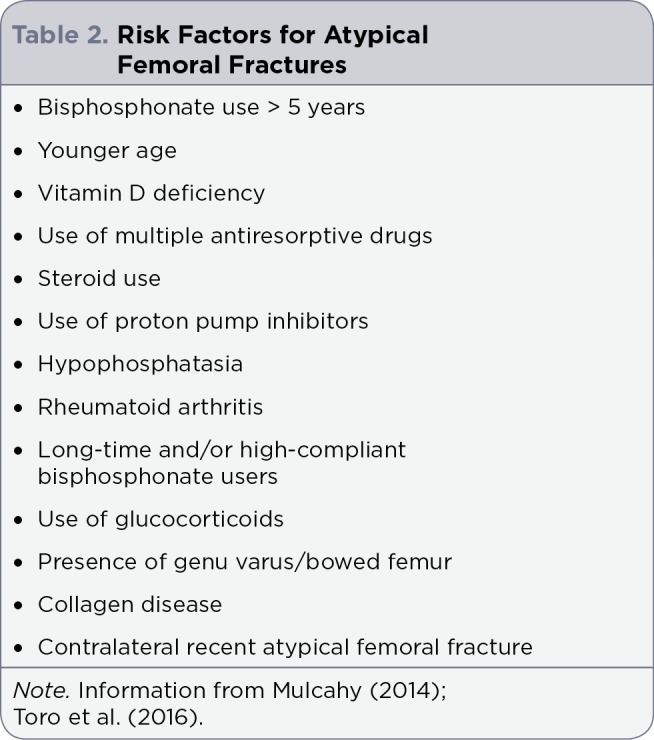
Risk Factors for Atypical Femoral Fractures

Approximately two-thirds of all women with breast cancer are postmenopausal with hormone-sensitive tumors. For many years, aromatase inhibitors (AIs) have been part of the standard of care for the estrogen receptor–positive breast cancer patient, because they reduce breast cancer mortality in postmenopausal women. However, AIs promote more rapid bone loss. Bisphosphonates have been a mainstay of osteoporosis treatment and prevention, and along with the RANKL inhibitor, denosumab, have been utilized to reduce the bone loss induced by AIs. Therefore, this group of patients, who are seen frequently in the oncology setting, is at increased risk for AFF.

Male patients with reduced testosterone production, such as those treated with androgen-deprivation therapy or orchiectomy, have an increased risk for osteoporosis and are likely to have been treated with bisphosphonates or a RANKL inhibitor to prevent bone loss. Other patient populations with potential bone-health issues and an increased risk for osteoporosis and AFF would include those who have received radiation (especially to the long bones), those taking high doses of steroids, survivors of childhood cancer, and those who have had stem cell transplant (Davenport, 2015; OncoLink, 2013).

## PATIENT EDUCATION

What does this information mean to the AP and to patients being treated with bisphosphonates or RANKL inhibitors? First, the patient must be managed according to standard guidelines for osteoporosis or bony metastasis. Regardless of the type of treatment initiated, the patient’s education must include information about atypical fracture and the associated symptom of mid-thigh pain, with instructions to report this problem to the health-care provider. Patients should be reassured that the risk of AFF is very low, generally < 1%. Close surveillance of patients and assessment for prodromal complaints of thigh pain are essential at every visit, particularly if they have been on therapy for several years.

## ASSESSMENT

**Figure 2:**
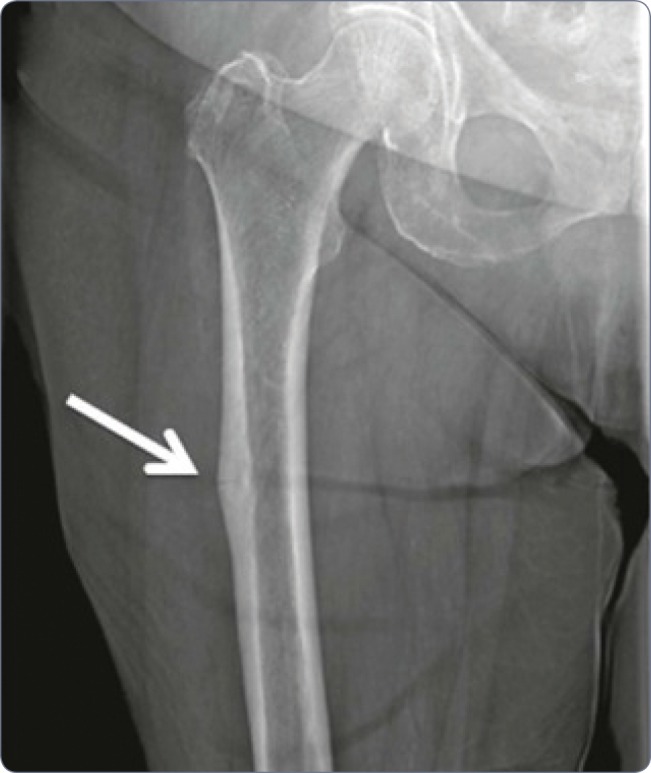
Radiograph of the right proximal femur showing an atypical, incomplete femoral diaphysis fracture (arrow) after long-term bisphosphonate therapy. Image adapted from Link and Adams (2016).

If a patient presents with groin pain or complains of mid-thigh pain, an x-ray of the affected femur should be performed, with a notation to the reading radiologist regarding concern for development of an AFF in a patient on bisphosphonates or RANKL inhibitors. If abnormalities are present on the x-ray of the affected leg, it is appropriate to order an x-ray of the opposite femur, since 30% of patients with an AFF have bilateral involvement. If the patient has no obvious AFF but does have continued pain, a computed tomography or magnetic resonance image may be needed to establish the presence of an AFF (Figure 2; Adler, Fuleihan, & Bauer, 2016; Toro et al., 2016; Tyler, Bakata, & O’Keefe, 2014; Mulcahy, 2014).

## MANAGEMENT

Minimally symptomatic patients with incomplete fractures or severe comorbidities precluding surgery can be given conservative treatment with a 3-month trial of no-weight bearing, but there is little evidence of the success of this approach. Several case reports have shown teriparatide (Forteo) to be beneficial, with reduced healing time and an increased union rate. However, the health-care provider should be mindful that teriparatide is contraindicated in patients with skeletal malignancy, bony metastasis, and metabolic bone disease (Adler et al., 2016; Toro et al., 2016; Tyler et al., 2014; Mulcahy, 2014).

The presence of a complete AFF generally calls for surgical repair, with intramedullary nailing as the surgical treatment of choice. When referring to surgery, APs should request that microscopic bone pathology and an assessment of fracture pattern be obtained at the time of surgery (Adler et al., 2016; Toro et al., 2016; Tyler et al., 2014; Mulcahy, 2014).

With every patient who experiences an AFF, evaluate/reevaluate the history for potential association with bisphosphonates and/or RANKL inhibitors. Treatment with these agents should be discontinued regardless if the fracture is incomplete or complete. Patients should be evaluated for adequate calcium and vitamin D intake, as well as the presence of any underlying and previously undiagnosed disease process (Adler et al., 2016; Toro et al., 2016; Tyler et al., 2014; Mulcahy, 2014).

## OTHER RECOMMENDATIONS

Oncology clinicians are utilizing bisphosphonates and denosumab to improve the bone health of patients; however, reports of AFFs have created uncertainty about the duration of bisphosphonate therapy for bone health in both the cancer and osteoporosis patient. The American Society for Bone and Mineral Research Task Force offers the following recommendations:

- After 5 years of oral bisphosphonate or 3 years of intravenous bisphosphonates, reassess risk.
- Consider continuing oral therapy for 10 years and intravenous therapy for 6 years if the fracture risk is high or the patient has had a fracture while on therapy.
- Consider a drug holiday with reassessment after 2 years, with resumption of therapy if indicated.
- Consider dose modification in patients who are continuing on daily corticosteroids, as risk may outweigh benefit.

For patients taking denosumab, the oncology provider should assess regularly for prodromal complaints of thigh pain. Unlike bisphosphonates, denosumab does not have a recommended limit on the duration of treatment. The FREEDOM extension trial, in which patients had up to 10 years of treatment, showed long-term safety with no increased incidence of an AFF over time (Chang et al., 2012; Adler et al., 2016; Papapoulous et al., 2015; Tyler et al., 2014; Mulcahy, 2014).

Decision-making in these cases can be difficult, but with a clear understanding of AFFs, the AP can confidently assess and care for patients on bone therapy should an AFF occur.
